# Synergies of fuel cell system thermal management and cryogenic hydrogen exergy utilization

**DOI:** 10.1038/s41598-022-26561-9

**Published:** 2022-12-21

**Authors:** Magnus Lenger, Steffen Heinke, Wilhelm Tegethoff, Jürgen Köhler

**Affiliations:** 1 Technische Universität Braunschweig, Institut für Thermodynamik, 38106 Braunschweig, Germany; 2 TLK-Thermo GmbH, 38106 Braunschweig, Germany

**Keywords:** Devices for energy harvesting, Fuel cells

## Abstract

Low-temperature polymer electrolyte fuel cell systems (FCSs) need to reject large amounts of low temperature heat. Often a mobile FCS’s cooling capacity limits the FCS power output. Cryogenic hydrogen is typically utilized as a direct heat sink using heat exchangers (HXs), even though HXs destroy most hydrogen exergy. This paper investigates synergies between FCS thermal management and cryogenic hydrogen exergy utilization in terms of their benchmark performance: the FCS coolant circuit supplies heat at coolant temperature level to a so named reversible cryogenic exergy utilization system (rCEUS) comprised of thermodynamically ideal heat engine processes. The rCEUS converts this heat partly to electrical energy (the value of which equals the hydrogen exergy) and rejects remaining heat to hydrogen to heat it to coolant temperature. The rCEUS output power is used to support the FCS, so the FCS rejects less heat and a significant fraction of this heat is utilized by the rCEUS. As a result, significantly less heat has to be transferred to ambient and the fuel demand decreases. In this paper, three hydrogen storage options are compared: liquid hydrogen, subcooled liquid hydrogen and cryo-compressed hydrogen. Different para- and orthohydrogen compositions are evaluated. For typical FCS operating points, rejected FCS heat to ambient is reducible by 40–67%. FCS power demand is reducible by 14–31%. FCS rejected heat to ambient reduction is 4.5–8 times larger than that of conventional HXs. Calculations are based on hydrogen’s lower heating value.

## Introduction

**Figure 1:**
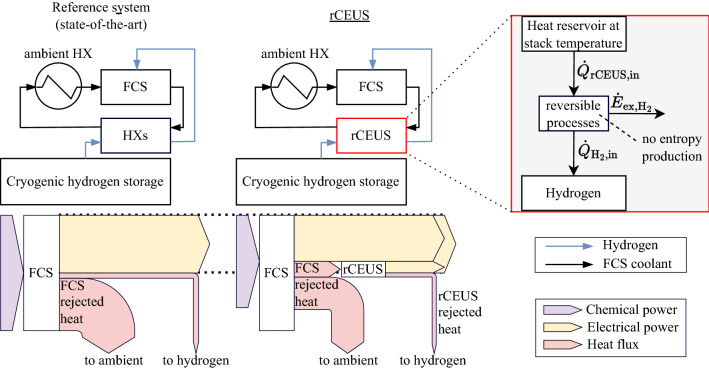
Conceptual illustration of a reversible hydrogen cryogenic exergy utilization system (rCEUS) in comparison to the state-of-the-art method of hydrogen conditioning for a fuel cell system (FCS) using heat exchangers (HXs). A simplified energy flow illustration is given for the case that the electrical system power output is fixed. rCEUS heat flows ${\dot{Q}}$ and rCEUS electrical power output ${\dot{E}}_{\mathrm {ex,H_2}}$, which is assumed equal to the hydrogen exergy flow, are shown.

In the light of contemporary anthropogenic climate changes with their present and future consequences a number of governments and institutions agreed upon drastic greenhouse gas emission reduction goals. On a global scale, there is for instance, the Paris Agreement of 2016^1^. Individual countries have also established domestic goals. The German government, for instance, aims for a reduction of greenhouse gas emissions by 80–85% compared to 1990 by 2050^2^. Using green hydrogen in fuel cell systems (FCSs) to generate electricity is one possibility for avoiding fossil fuels in certain sectors^3–6^. Among the different storage options of hydrogen in its pure form, cryogenic storages offer advantageous volumetric energy densities. However, bringing hydrogen (or any substance) to a cryogenic state requires energy. Part of this energy (ideally, the exergy) could be recovered. Technical applications for exergy recovery of cryogenic substances can be found mostly in liquefied natural gas regasification^7–15^, but also for example for liquid nitrogen^16^. For hydrogen^17, 18^, technical implementations are rarely considered and thermodynamically ideal cryogenic hydrogen exergy utilization systems as well as hydrogen conditioning systems for FCSs in general are currently (to the best knowledge of the authors) not considered in literature. At the same time, the thermal management of low-temperature polymer electrolyte FCSs poses challenges due to the low temperature level of heat rejection and the significant amount of rejected heat^19, 20^. Removing all rejected heat at FCS operating temperature in a limited installation space and with possibly minimal heat exchanger (HX) weight is often problematic. As a consequence, the available cooling capacity can limit the FCS electrical power output. The state-of-the-art solution for the thermal integration of a cryogenic storage system into FCSs are HXs that use FCS coolant to heat the required amount of hydrogen to the FCS operating temperature (see Fig. 1, top left image). Hydrogen exergy however, is mostly destroyed in HXs, while remaining exergy is typically destroyed in downstream throttles. Cryogenic hydrogen exergy in FCSs is an unexplored potential at the present day. Furthermore, the combination of hydrogen exergy utilization and the thermal management of fuel cell systems has not yet been subject to research. This paper thus presents a novel concept for cryogenic hydrogen conditioning for FCSs comprised of three functionalities: cryogenic hydrogen exergy is utilized to (i) generate auxiliary electrical power to support the FCS and (ii) decrease the required cooling capacity of the FCS thermal management system while (iii) conditioning hydrogen to fuel cell stack temperature. This paper’s objective is to quantify the concept’s thermodynamic benchmark performance in terms of FCS electrical power demand reduction, FCS rejected heat utilization and FCS rejected heat to ambient reduction (required cooling capacity reduction). Therefore, thermodynamically perfect, i.e. reversible heat engine processes between the constant FCS coolant temperature and the variable hydrogen temperature between hydrogen storage and fuel cell stack anode inlet are considered. No entropy is produced and the entire hydrogen exergy is utilized during hydrogen conditioning from cryogenic storage to fuel cell stack inlet state. These processes take place in a thermodynamically perfect machine, a so named reversible cryogenic exergy utilization system (rCEUS). The rCEUS topology and technical implementation of these processes is not subject of this paper; the rCEUS is treated as a black box while the focus lies on quantifying the thermodynamic potential of this technology. The FCS coolant circuit supplies heat to the rCEUS at coolant temperature level. The rCEUS converts this heat reversibly partly to electrical energy (the value of which equals the hydrogen exergy) and rejects the remaining heat at variable hydrogen temperature levels to heat the hydrogen to fuel cell stack anode inlet temperature. For all exergy calculations the reference state is therefore defined by the FCS stack temperature (which is approximately the averaged FCS coolant temperature) and fuel cell stack anode inlet pressure. The electrical power generated supplies part of the overall system’s output power such that the FCS output power and, thus, its rejected heat flux can be reduced. Therefore, by using the rCEUS, less chemical power has to be supplied to the FCS (fuel is saved), the amount of rejected FCS heat is thereby reduced, and parts of this remaining rejected heat is utilized (partially to generate electrical power and partially for hydrogen conditioning). This is shown in Fig. 1, where both conventional cryogenic hydrogen conditioning using HXs (left column) as well as the rCEUS concept (right column) with exemplary energy flow diagrams are depicted. Remaining FCS rejected heat that still has to be transferred to ambient is significantly reduced when deploying the rCEUS, as (1) the rCEUS uses more FCS rejected heat than HXs and (2) the amount of FCS rejected heat to begin with is reduced as the FCS electrical power demand is reduced.

In this work, the performance of this synergy will be quantified using FCS rejected heat reduction, rejected heat utilization and FCS electrical power reduction (see Fig. 1). Furthermore, the obtainable power and corresponding heat flows are assigned to hydrogen conditioning process steps.

## Methods

This sections elaborates on rCEUS energy exchange (heat in, heat out, work out) in comparison to heating hydrogen in HXs. Energy is assigned to different cryogenic hydrogen conditioning steps. The power outputs of FCS and rCEUS are coupled at constant FCS efficiency. All calculations are carried out based on steady-state processes. The rCEUS operates reversibly, i.e. it utilizes the entire hydrogen exergy and converts it to electrical energy without producing entropy.

### Specific heat demand for conditioning cryogenic hydrogen for fuel cell stacks

**Figure 2:**
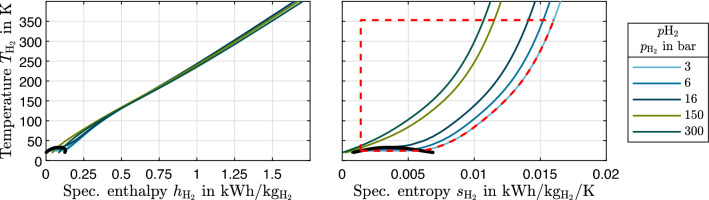
Parahydrogen isobars in a temperature-enthalpy and temperature-entropy diagram. The dashed red line indicates a reversible process that uses FCS rejected heat to generate work and to heat 3bar saturated liquid isobarically to 353K.

The mass-specific heat required to heat cryogenic hydrogen from its storage state to a desired FCS inlet state is

1 $$\begin{aligned} q_{\mathrm {H_2,in}} = h_{\mathrm {H_2}}(p_{\mathrm {FCS}},T_{\mathrm {FCS}},\xi _{\mathrm {FCS}}) - h_{\mathrm {H_2}}(p_{\mathrm {storage}},T_{\mathrm {storage}},\xi _{\mathrm {storage}}), \end{aligned}$$

with specific heat *q* and specific enthalpy *h*. In this paper, pressure *p*, temperature *T* and parahydrogen mass fraction $\xi$ are used as independent variables to define the thermodynamic state of hydrogen. In the vapor-liquid region, the vapor quality is also required, but this is not discussed here. As an example for the heat demand, increasing the temperature of saturated hydrogen vapor to 80 °C requires more than 10 times the evaporation enthalpy for pressures equal to or above 1bar (Fig. 2, left image).

**Figure 3:**
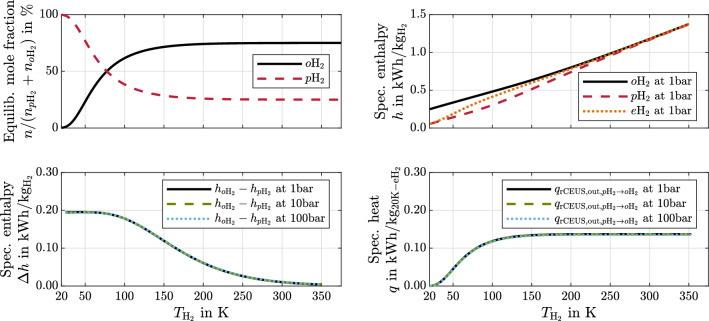
Top left: mole fractions of $o\mathrm {H}_2$ and $p\mathrm {H}_2$ in $e\mathrm {H}_2$. Top right: specific enthalpies of $o\mathrm {H}_2$, $p\mathrm {H}_2$ and $e\mathrm {H}_2$. Bottom left: conversion enthalpy from $p\mathrm {H}_2$ to $o\mathrm {H}_2$. Bottom right: heat for maintaining $e\mathrm {H}_2$ composition from 20K-$e\mathrm {H}_2$ to $T_{\mathrm {H_2}}$.

Molecular hydrogen is a mixture of its two forms parahydrogen (*p*H$_2$) and orthohydrogen (*o*H$_2$) that differ in their nuclear spin state configuration. There is a temperature dependent equilibrium composition called *e*H$_2$. At standard conditions and further increasing temperatures, the *e*H$_2$ composition contains around 25% *p*H$_2$ and 75% *o*H$_2$. A mixture with this composition is called normal hydrogen (*n*H$_2$). When approaching 0K, the *p*H$_2$ fraction in *e*H$_2$ approaches 100% (see Fig. 3 and the corresponding section later on). The *o*H$_2 \rightarrow$ *p*H$_2$ conversion is exothermic and is usually being catalyzed during liquefaction to avoid a delayed conversion in the liquid in order to minimize boil-off losses. As a result, cryogenic hydrogen is often composed mostly of *p*H$_2$ (the *p*H$_2$ content of 20K-*e*H$_2$ is 99.8%). The back reaction *p*H$_2 \rightarrow$ *o*H$_2$ is endothermic and requires around 0.14 kWh kg$^{-1}$ to continuously convert 20K-*e*H$_2$ back to the *n*H$_2$ composition. This is around 1.15 times the evaporation enthalpy at 1bar, around 10% of the enthalpy difference between the saturated liquid temperature and 80 $^{\circ }$C at 1bar, and 0.4% of the lower heating value of hydrogen of 33.3 kWh kg$^{-1}$.

There are different technical methods to supply the energy to heat hydrogen to FCS temperature, e.g. electric heating, the direct use of ambient heat or the direct use of FCS rejected heat. These approaches require energy while hydrogen exergy would in all cases be mostly (except for obtainable work from expansion to anode inlet pressure) destroyed. Instead, one might use processes which use FCS rejected heat as a heat source and hydrogen as a cold sink to heat hydrogen up to stack temperature and generate work. By doing so, one could remove more FCS rejected heat than the enthalpy difference and convert parts of it to work. The maximally obtainable work is the exergy. Exemplarily, heats and work of a reversible process that uses 353 K FCS rejected heat as a heat source and 3 bar saturated liquid hydrogen as a cold sink can be read from the process indicated by the dashed red line in Fig. 2, right image. The amount of utilizable FCS rejected heat is the area under the 353K-isotherm, the energy for hydrogen heating is the area under the isobar and the obtainable work (the exergy) corresponds to the enclosed area.

### Exergy of cryogenic hydrogen, corresponding amounts of heat and rCEUS energy balance

The exergy of a substance depends on its state and on an available thermodynamic reference state^21^. In this work, the reference state is defined by a desired hydrogen pressure and temperature at the FCS inlet (index ‘FCS’). This is done because there is typically more FCS rejected heat at stack operating temperature available than maximally utilizable by a hydrogen rCEUS. With the cryogenic storage state described by pressure and temperature, the specific exergy with respect to the FCS inlet is

2 $$\begin{aligned} e_{\mathrm {ex, H_2}}= & T\Delta s - \Delta h = w_{\mathrm {rCEUS,out}} \nonumber \\= & T_{\mathrm {FCS}}\cdot (s_{\mathrm {H_2}}(p_{\mathrm {FCS}},T_{\mathrm {FCS}},\xi _{\mathrm {FCS}}) - s_{\mathrm {H_2}}(p_{\mathrm {storage}},T_{\mathrm {storage}},\xi _{\mathrm {storage}})) \nonumber \\&\quad - h_{\mathrm {H_2}}(p_{\mathrm {FCS}},T_{\mathrm {FCS}},\xi _{\mathrm {FCS}}) + h_{\mathrm {H_2}}(p_{\mathrm {storage}},T_{\mathrm {storage}},\xi _{\mathrm {storage}}), \end{aligned}$$

with specific exergy $e_{\mathrm {ex}}$ and specific entropy *s* of the considered $p\mathrm {H}_2$–$o\mathrm {H}_2$ mixture, the $p\mathrm {H}_2$ mass fraction $\xi$ and the specific work *w*. Since the hydrogen exergy depends on the $p\mathrm {H}_2$–$o\mathrm {H}_2$ composition, different mixtures are considered: cryogenic hydrogen as $n\mathrm {H}_2$, as $p\mathrm {H}_2$ and as $e\mathrm {H}_2$, i.e. hydrogen that is continuously converted to equilibrium composition during temperature increase.

To assess which hydrogen processing step accounts for which exergy fraction, the exergy is expressed as a sum of maximally obtainable work from each processing step. Those steps are: sensible heating of subcooled liquid, evaporation, sensible gas heating (supercritical hydrogen included), *p*H$_2 \rightarrow o$H$_2$ conversion and expansion (solid hydrogen is excluded), such that

3 $$\begin{aligned} e_{\mathrm {ex, H_2}}= & w_{\mathrm {rCEUS,out}} = w_{\mathrm {sensible \; liquid \; heating, max}} + w_{\mathrm {evaporation, max}} + w_{\mathrm {sensible \; gas \; heating, max}} \nonumber \\&\quad + w_{p\mathrm {H}_2 \rightarrow o\mathrm {H_2, max}} + w_{\mathrm {expansion, max}}. \end{aligned}$$

The magnitude of each term in Eq. (3) is defined by the process path. For instance, starting from the cryogenic storage state, one might use a reversible adiabatic expansion to reference pressure and subsequent isobaric heating to reference temperature, or start with isobaric heat supply to reference temperature and a subsequent isothermal expansion to reference pressure.

The maximum specific heat that can be supplied to a rCEUS is:

4 $$\begin{aligned} q_{\mathrm {rCEUS,in}} =T_{\mathrm {FCS}}\cdot (s_{\mathrm {H_2}}(p_{\mathrm {FCS}},T_{\mathrm {FCS}},\xi _{\mathrm {FCS}}) - s_{\mathrm {H_2}}(p_{\mathrm {storage}},T_{\mathrm {storage}},\xi _{\mathrm {storage}})). \end{aligned}$$

The maximum specific rCEUS heat input can also be expressed as the sum of the specific heat inputs of each process step:

5 $$\begin{aligned} q_{\mathrm {rCEUS,in}} &= q_{\mathrm {rCEUS,in,sensible \; liquid \; heating, max}}+ q_{\mathrm {rCEUS,in,evaporation, max}} + q_{\mathrm {rCEUS,in,sensible \; gas \; heating, max}} \nonumber \\ &\quad+ q_{\mathrm {rCEUS,in,}p\mathrm {H}_2 \rightarrow o\mathrm {H}_2,\mathrm {max}} + q_{\mathrm {rCEUS,in,expansion, max}}. \end{aligned}$$

The reversible process’s energy balance (Fig. 1) can then be written with Eqs. (4), (1) and (3) and hydrogen mass flow ${\dot{m}}_{\mathrm {H_2}}$:

6 $$\begin{aligned} 0 = {\dot{Q}}_{\mathrm {rCEUS,in}} - {\dot{Q}}_{\mathrm {H_2,in}} - {\dot{E}}_{\mathrm {ex, H_2}} = {\dot{m}}_{\mathrm {H_2}}\cdot (q_{\mathrm {rCEUS,in}} - q_{\mathrm {H_2,in}} - e_{\mathrm {ex, H_2}}) \;\;\;\; \mathrm {where} \;\;\;\; q_{\mathrm {H_2,in}} = q_{\mathrm {rCEUS,out}}. \end{aligned}$$

### Maximally obtainable work from cryogenic hydrogen and corresponding heat supply and rejection

The maximum work obtainable from each mentioned process step can be determined with a second law analysis. The entire obtainable work can only be utilized if the process that brings the hydrogen to equilibrium with the reference state (index ‘FCS’) produces no entropy. The hydrogen mass specific rCEUS entropy balance yields (note that $q_{\mathrm {H_2,in}} = q_{\mathrm {rCEUS,out}}$):

7 $$\begin{aligned} \frac{\mathrm {\delta } q_{\mathrm {rCEUS,in}}}{T_{\mathrm {FCS}}} - \frac{\mathrm {\delta } q_{\mathrm {H_2,in}}}{T_{\mathrm {H_2}}} = 0 \;\;\;\; \mathrm {with} \;\;\;\; T_{\mathrm {FCS}} = \mathrm {constant}. \end{aligned}$$

Here, $q_{\mathrm {rCEUS,in}}$ is the specific heat supplied to the rCEUS at FCS coolant temperature level $T_{\mathrm {FCS}}$ from the coolant circuit. $q_{\mathrm {H_2,in}}$ is the rCEUS specific rejected heat. This is the heat supplied to the hydrogen at its prevalent temperature $T_{\mathrm {H_2}}$.

Together with the first law of thermodynamics, the maximum heat supply and the corresponding maximally obtainable work is

8 $$\begin{aligned} \mathrm {\delta } q_{\mathrm {rCEUS,in}}&= \frac{T_{\mathrm {FCS}}}{T_{\mathrm {H_2}}} \mathrm {\delta } q_{\mathrm {rCEUS,out}} = \frac{T_{\mathrm {FCS}}}{T_{\mathrm {H_2}}} \mathrm {\delta } q_{\mathrm {H_2,in}}, \end{aligned}$$

9 $$\begin{aligned} \mathrm {\delta } w_{\mathrm {rCEUS,out}}&= \left( \frac{T_{\mathrm {FCS}}}{T_{\mathrm {H_2}}} -1 \right) \mathrm {\delta } q_{\mathrm {rCEUS,out}} = \left( \frac{T_{\mathrm {FCS}}}{T_{\mathrm {H_2}}} -1 \right) \mathrm {\delta } q_{\mathrm {H_2,in}}. \end{aligned}$$

#### Maximally obtainable work from isobaric evaporation and isobaric sensible heating of cryogenic hydrogen

The evaporation (heat supply at constant temperature) has to be differentiated from sensible heating (heat supply at varying temperature). For isobaric evaporation, the maximally obtainable work and specific heats involved are then simply determined with the pressure dependent vaporization enthalpy $h_{\mathrm {H_2}}'' - h_{\mathrm {H_2}}'$ of the considered *p*H$_2$–*o*H$_2$ mixture:

10 $$\begin{aligned} q_{\mathrm {rCEUS, in, evaporation}}&= q_{\mathrm {rCEUS, out, evaporation}} \frac{T_{\mathrm {FCS}}}{T_{\mathrm {H_2}}}, \end{aligned}$$

11 $$\begin{aligned} q_{\mathrm {rCEUS, out, evaporation}}&= h_{\mathrm {H_2}}''(p_{\mathrm {H_2}}) - h_{\mathrm {H_2}}'(p_{\mathrm {H_2}}) = q_{\mathrm {H_2,in,evaporation}}, \end{aligned}$$

12 $$\begin{aligned} w_{\mathrm {rCEUS, out, evaporation}}&= \left( h_{\mathrm {H_2}}''(p_{\mathrm {H_2}}) - h_{\mathrm {H_2}}'(p_{\mathrm {H_2}}) \right) \cdot \left( \frac{T_{\mathrm {FCS}}}{T_{\mathrm {H_2}}} -1 \right) . \end{aligned}$$

The maximally obtainable work and corresponding heats (in and out of the rCEUS) from sensible heating of liquid as well as gaseous or supercritical hydrogen isobarically and reversibly from some temperature $T_{\mathrm {0}}$ to $T_{\mathrm {1}}$ are

13 $$\begin{aligned} q_{\mathrm {rCEUS, in, sensible \; heating}}& = T_{\mathrm {FCS}} \int _{T_{\mathrm {0}}}^{T_{\mathrm {1}}} \frac{1}{T_{\mathrm {H_2}}} \frac{\partial h_{\mathrm {H_2}}(p_{\mathrm {H_2}},T_{\mathrm {H_2}},\xi _{\mathrm {H_2}}(T_{\mathrm {H_2}}))}{\partial T_{\mathrm {H_2}}} \mathrm {d}T_{\mathrm {H_2}}, \end{aligned}$$

14 $$\begin{aligned}&= T_{\mathrm {FCS}} \cdot \left( s_{\mathrm {H_2}}(p_{\mathrm {H_2,0}},T_{\mathrm {H_2,1}},\xi _{\mathrm {H_2,1}})-s_{\mathrm {H_2}}(p_{\mathrm {H_2,0}},T_{\mathrm {H_2,0}},\xi _{\mathrm {H_2,0}}) \right) , \end{aligned}$$

15 $$\begin{aligned} q_{\mathrm {rCEUS, out, sensible \; heating}}&= h_{\mathrm {H_2}}(p_{\mathrm {H_2,0}},T_{\mathrm {H_2,1}},\xi _{\mathrm {H_2,1}})-h_{\mathrm {H_2}}(p_{\mathrm {H_2,0}},T_{\mathrm {H_2,0}},\xi _{\mathrm {H_2,0}}) = q_{\mathrm {H_2, in, sensible \; heating}}, \end{aligned}$$

16 $$\begin{aligned} w_{\mathrm {rCEUS, out, sensible \; heating}}&= q_{\mathrm {rCEUS, in, sensible \; heating}} - q_{\mathrm {rCEUS, out, sensible \; heating}}. \end{aligned}$$

#### Maximally obtainable work and heat flows involved in an isobaric *p*H$_2 \rightarrow o$H$_2$ spin state conversion

Considering a hydrogen mixture at a temperature below that of the FCS stack with an *o*H$_2$ fraction below that of *e*H$_2$, one can increase the exergy by utilizing the *p*H$_2 \rightarrow o$H$_2$ spin state conversion, i.e. by increasing the *o*H$_2$ fraction during sensible hydrogen heating. This is, because the *p*H$_2 \rightarrow o$H$_2$ conversion is endothermic and occurs at temperatures below the reference temperature. By doing so, one can utilize more rejected FCS heat. The maximally obtainable work and corresponding amounts of heat from the spin state conversion can be determined with

1. the hydrogen state dependent specific conversion enthalpy: $\Delta h_{p\mathrm {H}_2 \rightarrow o\mathrm {H}_2}(p,T)$
2. the temperature dependent mole fraction (= mass fraction) of convertible *p*H$_2$: $\xi _{p\mathrm {H}_2}(T)$

The specific conversion enthalpy is $\Delta h_{p\mathrm {H}_2 \rightarrow o\mathrm {H}_2} = h_{o\mathrm {H_2}}(p,T) - h_{p\mathrm {H_2}}(p,T)$. It can be determined with adequately normalized enthalpy equations of state^22^ for both forms. The enthalpies are illustrated in the top right image of Fig. 3.

The equilibrium composition is shown in the top left image. It obeys a Boltzmann distribution^23^ and can be calculated using

17 $$\begin{aligned} \frac{n_{p\mathrm {H_2}}}{n_{o\mathrm {H_2}}} = \frac{\sum _{i=0,2,4,...}(2i+1) \mathrm {e}^{\frac{-i(i+1)\Theta _{\mathrm {H_2}}}{T_{\mathrm {H_2}}} }}{\sum _{j=1,3,5,...}3(2j+1)\mathrm {e}^{\frac{ -j(j+1)\Theta _{\mathrm {H_2}}}{T_{\mathrm {H_2}}}}} \;\;\;\; \;\;\;\; \mathrm {with} \;\;\;\; \;\;\;\; \Theta _{\mathrm {H_2}} = \frac{\hslash ^2}{2 I_{\mathrm {H_2}} k_{\mathrm {B}}} = {86.2}K \;\;\;\; \;\;\;\; \mathrm {and} \;\;\;\; \;\;\;\; n_{p\mathrm {H_2}} + n_{o\mathrm {H_2}} = n_{\mathrm {H_2}}. \end{aligned}$$

In Eq. (17), *n* denotes the amount of substance. $\Theta _{\mathrm {H_2}}$ is hydrogen’s rotational constant. It is determined with hydrogen’s moment of inertia $I_{\mathrm {H_2}} = 4.67 \times 10^{-48}$ kg m$^{2}$, the reduced Planck constant $\hslash = 1.055 \times 10^{-34}$ Js and Boltzmann’s constant $k_{\mathrm {B}} = 1.381 \cdot 10^{-23}$ JK$^{-1}$. The $p\mathrm {H}_2$ fraction in $e\mathrm {H}_2$ is a function of temperature only and becomes with the above equations

18 $$\begin{aligned} \xi _{p\mathrm {H}_2} := \frac{n_{p\mathrm {H_2}}}{n_{p\mathrm {H_2}}+n_{o\mathrm {H_2}}} = \left( 1 + \frac{\sum _{j=1,3,5,...}3(2j+1)\mathrm {e}^{\frac{ -j(j+1)\Theta _{\mathrm {H_2}}}{T_{\mathrm {H_2}}}}}{\sum _{i=0,2,4,...}(2i+1) \mathrm {e}^{\frac{-i(i+1)\Theta _{\mathrm {H_2}}}{T_{\mathrm {H_2}}} }} \right) ^{-1} = \xi _{p\mathrm {H_2}}(T_{\mathrm {H_2}}). \end{aligned}$$

The amount of convertible *p*H$_2$ during a temperature increase by some $\mathrm {d}T$ is $\mathrm {d} \xi _{p\mathrm {H}_2}$. To determine work and amounts of heat involved in a reversible conversion for a certain temperature interval, one can use the total derivative of $\xi _{p\mathrm {H_2}}$: $\mathrm {d}\xi _{p\mathrm {H_2}} = \frac{\mathrm {d} \xi _{p\mathrm {H_2}}(T_{\mathrm {H_2}})}{\mathrm {d} T_{\mathrm {H_2}}} \mathrm {d}T_{\mathrm {H_2}}$. Based on Eqs. (8) and (9), the maximally obtainable work and corresponding amounts of heat (in and out of the rCEUS) from an isobaric and reversible *p*H$_2 \rightarrow o$H$_2$ conversion then become

19 $$\begin{aligned} q_{\mathrm {rCEUS, in,} p\mathrm {H}_2 \rightarrow o\mathrm {H_2}}= & - T_{\mathrm {FCS}}\int _{T_{\mathrm {0}}}^{T_{\mathrm {1}}} \frac{1}{T_{\mathrm {H_2}}} \Delta h_{p\mathrm {H}_2 \rightarrow o\mathrm {H}_2} (T_{\mathrm {H_2}},p_{\mathrm {H_2}}) \frac{\mathrm {d} \xi _{p\mathrm {H_2}}(T_{\mathrm {H_2}})}{\mathrm {d} T_{\mathrm {H_2}}} \mathrm {d}T_{\mathrm {H_2}} \;\;\;\; \;\;\;\; \mathrm {with} \;\;\;\; \;\;\;\; p_{\mathrm {H_2}} = \mathrm {constant}, \end{aligned}$$

20 $$\begin{aligned} q_{\mathrm {rCEUS, out,} p\mathrm {H}_2 \rightarrow o\mathrm {H}_2}&= - \int _{T_{\mathrm {0}}}^{T_{\mathrm {1}}} \Delta h_{p\mathrm {H}_2 \rightarrow o\mathrm {H}_2} (T_{\mathrm {H_2}},p_{\mathrm {H_2}}) \frac{\mathrm {d} \xi _{p\mathrm {H_2}}(T_{\mathrm {H_2}})}{\mathrm {d} T_{\mathrm {H_2}}} \mathrm {d}T_{\mathrm {H_2}}, \;\;\;\; \;\;\;\; \;\;\;\; \;\;\;\;\;\;\;\;\;\;\;\; \mathrm {with} \;\;\;\; \;\;\;\; p_{\mathrm {H_2}} = \mathrm {constant} \end{aligned}$$

21 $$\begin{aligned} w_{\mathrm {rCEUS, out,} p\mathrm {H}_2 \rightarrow o\mathrm {H}_2}&= q_{\mathrm {rCEUS, in,} p\mathrm {H}_2 \rightarrow o\mathrm {H_2}} - q_{\mathrm {rCEUS, out,} p\mathrm {H}_2 \rightarrow o\mathrm {H}_2}. \end{aligned}$$

Given that *p*H$_2$ and *o*H$_2$ have different specific heat capacities, the conversion enthalpy per kilogram *p*H$_2$ is temperature dependent (compare the top right and bottom left images of Fig. 3). Furthermore, the convertible amount of *p*H$_2$ also depends on temperature because of the *e*H$_2$ composition’s temperature dependency. This affects the maximally obtainable work and corresponding amounts of heat and is considered in Eqs. (19)–(21). Evaluating Eq. (20) shows how much heat the conversion requires up to a certain hydrogen temperature. The specific heat is shown in Fig. 3 (bottom right image) exemplarily for *e*H$_2$ at an initial temperature of 20K. The conversion heat is almost independent from pressure and around 1.15 times the evaporation enthalpy at 1 bar.

#### Maximally obtainable work and corresponding amounts of heat from isothermal expansion

To obtain the entire hydrogen exergy with respect to the reference state, a reversible expansion to FCS inlet pressure is required if the storage pressure is above that of the FCS inlet. Beginning from the storage state, one could either use isobaric heat supply to FCS temperature and subsequent isothermal expansion to FCS pressure, or adiabatic reversible expansion to FCS pressure and subsequent isobaric heat supply to FCS temperature. Isothermal expansion is used here. The maximally obtainable work and heats exchanged in isothermal expansion are

22 $$\begin{aligned} q_{\mathrm {rCEUS, in, isoth. exp}}&= T_{\mathrm {FCS}} \cdot (s_{\mathrm {H_2}}(p_{\mathrm {FCS}},T_{\mathrm {FCS}},\xi _{\mathrm {FCS}}) - s_{\mathrm {H_2}}(p_{\mathrm {storage}},T_{\mathrm {FCS}},\xi _{\mathrm {FCS}})), \end{aligned}$$

23 $$\begin{aligned} q_{\mathrm {rCEUS, out, isoth. exp}}&= 0, \end{aligned}$$

24 $$\begin{aligned} w_{\mathrm {rCEUS, out, isoth. exp}}&= q_{\mathrm {rCEUS, in, isoth. exp}} + h_{\mathrm {H_2}}(p_{\mathrm {storage}},T_{\mathrm {FCS}},\xi _{\mathrm {FCS}}) - h_{\mathrm {H_2}}(p_{\mathrm {FCS}},T_{\mathrm {FCS}},\xi _{\mathrm {FCS}}). \end{aligned}$$

### Hydrogen demand and rejected fuel cell system heat

Considering full hydrogen utilization in the FCS, the hydrogen mass flow ${\dot{m}}_{\mathrm {H_2}}$ for a certain electrical FCS power $P_{\mathrm {el, stack, out}}$ and the resulting rejected FCS heat flux ${\dot{Q}}_{\mathrm {FCS,out}}$ is

25 $$\begin{aligned} {\dot{m}}_{\mathrm {H_2}} = \frac{P_{\mathrm {chem, stack, in}}}{w_{\mathrm {H_2}}} = \frac{P_{\mathrm {el, stack, out}}}{\eta _{\mathrm {FCS}}w_{\mathrm {H_2}}} \;\;\;\; \;\;\;\; \mathrm {and} \;\;\;\; \;\;\;\; {\dot{Q}}_{\mathrm {FCS,out}} = P_{\mathrm {el, stack, out}} \cdot \left( \frac{1}{\eta _{\mathrm {FCS}}} -1 \right) , \end{aligned}$$

assuming gaseous water exhaust with the lower heating value of hydrogen $w_{\mathrm {H_2}} =$ 120 MJ kg$^{-1}$ and the FCS efficiency $\eta _{\mathrm {FCS}}$ that is the ratio of net electrical FCS power to chemical power $P_{\mathrm {chem, stack, in}}$ supplied to the stack via hydrogen. It is assumed that the entire FCS rejected heat flux is transferred to the FCS coolant circuit.

### Coupling the output powers of FCS and rCEUS

Given some fixed electrical power demand $P_{\mathrm {el, system, out}}$, the FCS of the reference system (Fig. 1, left side) supplies this demand entirely by itself. The rCEUS supplies a part of $P_{\mathrm {el, system, out}}$ to reduce the FCS load (Fig. 1, right side). This reduces the hydrogen demand and amount of rejected FCS heat. Less hydrogen mass flow leads to less rCEUS output power. This in turn influences the FCS load and so forth. These interdependencies are reflected in the system of linear equations that emerges:

26 $$\begin{aligned} P_{\mathrm {chem, stack, in}}= & \frac{P_{\mathrm {el, stack, out}}}{\eta _{\mathrm {FCS}}} \;\;\;\; \mathrm {where} \;\;\;\; \eta _{\mathrm {FCS}} = \mathrm {constant}, \end{aligned}$$

27 $$\begin{aligned} {\dot{m}}_{\mathrm {H_2}}= & \frac{P_{\mathrm {chem, stack, in}}}{w_{\mathrm {H_2}}}, \end{aligned}$$

28 $$\begin{aligned} P_{\mathrm {rCEUS, out}}= & {\dot{m}}_{\mathrm {H_2}} e_{\mathrm {ex, H_2}} \end{aligned}$$

29 $$\begin{aligned} P_{\mathrm {el, stack, out}}= & P_{\mathrm {el, system, out}} - P_{\mathrm {rCEUS, out}}. \end{aligned}$$

Here, $P_{\mathrm {rCEUS, out}}$ is rCEUS electrical output power. The solution corresponds to a state with less FCS rejected heat and a reduced FCS output power (compare also Fig. 1).

Four rCEUS evaluation parameters are introduced and highlighted in the bullet points that follow. These parameters are the:

- share of system output power the rCEUS can provide: $\Pi _P$ One can find a correlation between $\Pi _P$, the hydrogen storage state, and the specific hydrogen exergy:
  30 $$\begin{aligned} \Pi _P := \frac{\tilde{P}_{\mathrm {rCEUS, out}}}{P_{\mathrm {el, system, out}}} =\frac{\tilde{{\dot{m}}}_{\mathrm {H_2}}e_{\mathrm {ex,H_2}}}{P_{\mathrm {el, system, out}}} . \end{aligned}$$
  The tilde ( $\tilde{\cdot }$ ) indicates that rCEUS and FCS powers are coupled, i.e. that Eqs. (26)–(29) are solved.
- share of rejected FCS heat utilizable in the rCEUS: $\Pi _{{\dot{Q}}}$ With Eqs. (4) and (25) one can correlate $\Pi _{{\dot{Q}}}$, hydrogen storage state, specific hydrogen exergy, and FCS efficiency:
  31 $$\begin{aligned} \Pi _{{\dot{Q}}} := \frac{{\dot{Q}}_{\mathrm {rCEUS, in}}}{\tilde{{\dot{Q}}}_{\mathrm {FCS, out}}} = \frac{ T_{\mathrm {FCS}} \cdot (s_{\mathrm {H_2}}(p_{\mathrm {FCS}},T_{\mathrm {FCS}},\xi _{\mathrm {FCS}}) - s_{\mathrm {H_2}}(p_{\mathrm {storage}},T_{\mathrm {storage}},\xi _{\mathrm {storage}})) }{w_{\mathrm {H_2}} \cdot (1-\tilde{\eta }_{\mathrm {FCS}})}. \end{aligned}$$
  Here, ${\dot{Q}}_{\mathrm {rCEUS, in}}= {\dot{m}}_{\mathrm {H_2}} q_{\mathrm {rCEUS, in}}$ (Eq. 4). As a simplification, $\eta _{\mathrm {FCS}}$ is assumed constant despite FCS load changes.
- FCS rejected heat to ambient reduction including FCS power downsizing: $\Phi _{{\dot{Q}}}$ In case the FCS and rCEUS are coupled, meaning the reference power demand is partially provided by the rCEUS, the FCS power demand, the hydrogen mass flow and therefore its rejected heat flux decreases. Parts of the remaining heat flux are utilized by the rCEUS. $\Phi _{{\dot{Q}}}$ quantifies the rejected heat flux to ambient reduction of a reference FCS:
  32 $$\begin{aligned} \Phi _{{\dot{Q}}} := 1 - \frac{ \tilde{{\dot{Q}}}_{\mathrm {FCS, out}} - \tilde{{\dot{Q}}}_{\mathrm {rCEUS, in}} }{\hat{{\dot{Q}}}_{\mathrm {FCS,out}}}. \end{aligned}$$
  Heat fluxes are determined with Eqs. (4) and (25). The hat ( ${\hat{\cdot }}$ ) indicates quantities which are determined for the case in which the FCS provides the reference power demand all by itself, i.e. before a rCEUS is integrated. For example: $\Phi _{{\dot{Q}}} =$ 60% means, FCS rejected heat to ambient (its required cooling capacity) is reducible by 60% if coupled with a rCEUS.
- share of rejected FCS heat flux for heating hydrogen using HXs only: $\Pi _{{\dot{Q}},\mathrm {HX}}$
  33 $$\begin{aligned} \Pi _{{\dot{Q}},\mathrm {HX}} := \frac{{\dot{m}}_{\mathrm {H_2}}(h_{\mathrm {H_2,FCS}} - h_{\mathrm {H_2,storage}})}{{\dot{Q}}_{\mathrm {FCS,out}}} = \frac{h_{\mathrm {H_2}}(p_{\mathrm {FCS}},T_{\mathrm {FCS}},\xi _{\mathrm {FCS}}) - h_{\mathrm {H_2}}(p_{\mathrm {storage}},T_{\mathrm {storage}},\xi _{\mathrm {storage}})}{w_{\mathrm {H_2}} \cdot (1-{\hat{\eta }}_{\mathrm {FCS}})}. \end{aligned}$$
  To compare cooling capabilities of rCEUS and a reference system that uses HXs only, one can evaluate $\Phi _{{\dot{Q}}} / \Pi _{{\dot{Q}},\mathrm {HX}}$. For instance, $\Phi _{{\dot{Q}}} / \Pi _{{\dot{Q}},\mathrm {HX}} = 8$ means a rCEUS achieves 8 times the FCS rejected heat to ambient reduction HXs do.

## Results and discussion

**Figure 4:**
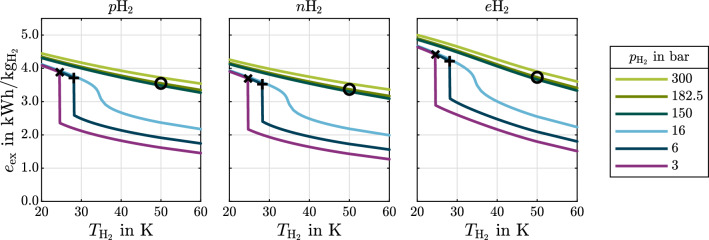
Specific exergy of cryogenic *p*H$_2$, *n*H$_2$ and *e*H$_2$. The reference state is defined by an averaged fuel cell system coolant temperature of 353 K and fuel cell stack inlet pressure of 2 bar. **x** $\widehat{=}$ LH$_2$, **+** $\widehat{=}$ sLH$_2$, **o** $\widehat{=}$ CcH$_2$.

**Figure 5:**
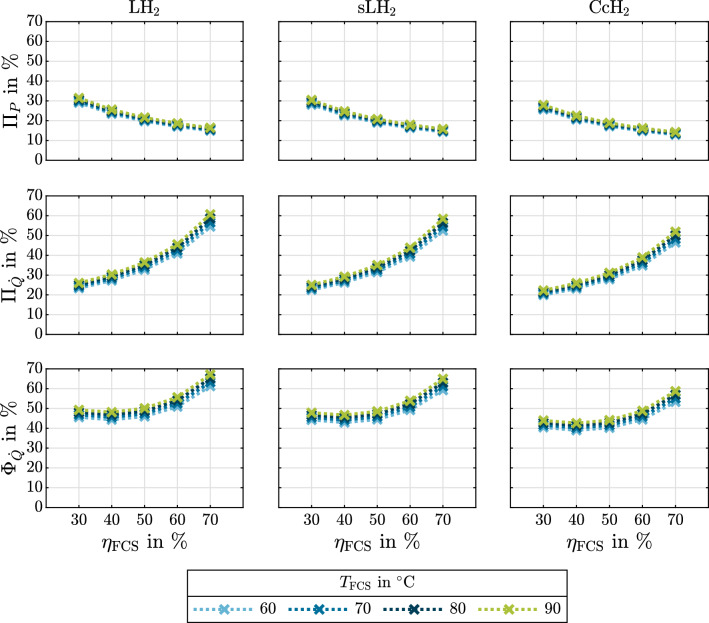
The rCEUS’s system output power share (top row), FCS rejected heat utilization (middle row) and FCS rejected heat to ambient reduction (bottom row) for *e*H$_2$ stored as LH$_2$ (3 bar, 24.6 K), sLH$_2$ (6 bar, 28.1 K) and CcH$_2$ (182.5 bar, 50 K) for varying FCS efficiencies and operating temperatures. The *p*H$_2 \rightarrow$ *o*H$_2$ conversion is utilized in all cases.

**Figure 6:**
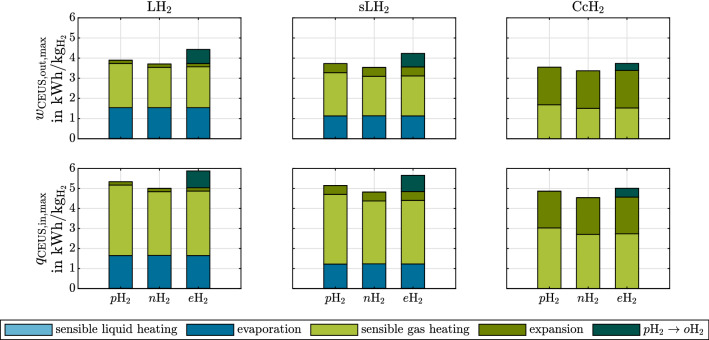
Maximally obtainable work (top row) and maximum utilizable FCS rejected heat (bottom row) for each process step of hydrogen conditioning when utilizing hydrogen exergy. Left column: LH$_2$ (3 bar saturated liquid), middle column: sLH$_2$ (6 bar saturated liquid), right column: CcH$_2$ (182.5 bar, 50 K). The reference state is a fuel cell stack inlet state of 2 bar, 353 K.

**Figure 7:**
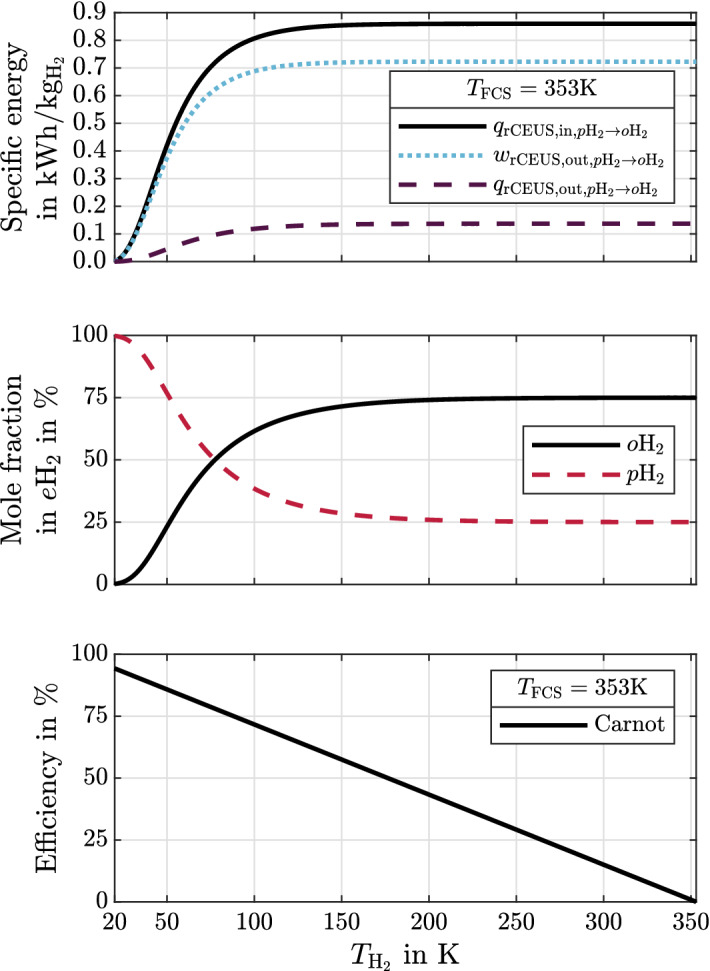
Top: Obtainable work and specific heats from a continuous *p*H$_2 \rightarrow o$H$_2$ conversion using 353 K FCS heat and the rCEUS: $e\mathrm {H}_2$ composition is maintained from initially 20 K to $T_{\mathrm {H_2}}$. Middle: *p*H$_2$ and *o*H$_2$ mole fractions in *e*H$_2$. Bottom: Carnot efficiency at every temperature $T_{\mathrm {H_2}}$ at constant $T_{\mathrm {FCS}}={353} K$.

**Figure 8:**
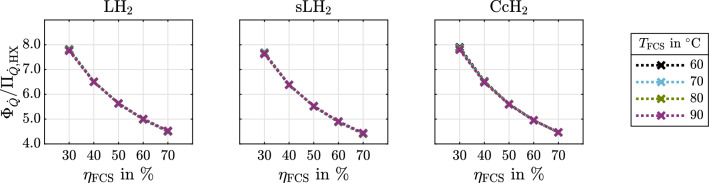
Ratio of FCS rejected heat reductions when using a rCEUS ($\Phi _{{\dot{Q}}}$) and HXs ($\Pi _{{\dot{Q}}, \mathrm {HX}}$). Cryogenic *e*H$_2$ is shown as LH$_2$ (3 bar, 24.6 K), sLH$_2$ (6 bar, 28.1 K) and CcH$_2$ (182.5 bar, 50 K) for varying FCS efficiencies and FCS operating temperatures.

The following results emphasize the physical significance of cryogenic hydrogen exergy utilization in fuel cell electric mobility. The presented diagrams can be used to quickly assess and quantify benchmark synergistic effects between some given cryogenic storage system and FCS. It is therefore shown how much exergy is contained in cryogenic hydrogen (Fig. 4). rCEUS induced FCS power demand reduction, subsequent rejected heat to ambient reduction and rejected heat utilization are shown in Fig. 5. The shares of rCEUS output power and utilized FCS rejected heat corresponding to each hydrogen conditioning process step are illustrated in Fig. 6 including the *p*H$_2 \rightarrow o$H$_2$ conversion. The implications of utilizing the *p*H$_2 \rightarrow o$H$_2$ conversion are discussed (Fig. 7). FCS rejected heat to ambient reduction achieved by the rCEUS is compared to conventional HXs (Fig. 8). Three hydrogen composition options (*p*H$_2$, *n*H$_2$ and *e*H$_2$) as well as the following three hydrogen storage states are considered:

- Liquid hydrogen (LH$_2$): saturated liquid at 3bar
- Subcooled liquid hydrogen (sLH$_2$): saturated liquid at 6bar
- Cryo-compressed hydrogen (CcH$_2$): supercritical hydrogen, averaged^24^ to 182.5 bar and 50 K.

### Specific exergy of cryogenic hydrogen

For *p*H$_2$, *n*H$_2$ and *e*H$_2$ Fig. 4 shows specific exergy versus temperature. The amount of exergy directly links to the fraction of required FCS output power that could be supplied by a rCEUS (Eq. 30) and correlates with the amount of rejected FCS heat that can be reduced via FCS electrical power output reduction.

The exergy-temperature diagram allows the following conclusions:

- within the vapor-liquid region, the lower the pressure, the higher the density and thus the higher the specific exergy.
- the maximum rCEUS output power at a certain FCS operating point can easily be determined using Eq. (30). This maximum power can be determined for different hydrogen storage states during operation.
- the obtainable rCEUS power can be assessed for the case that a rCEUS is installed only downstream a certain hydrogen state. For instance: if the latent heat of evaporation can not be utilized, one can determine the remaining obtainable power of the vapor from reading the specific exergy off the saturated vapor line at the corresponding pressure.
- the *e*H$_2$ exergy is up to 15% of hydrogen’s lower heating value of 33.3 kWh kg$^{-1}$ for CcH$_2$ at 300 bar, 20 K.
- fueling *p*H$_2$ instead of *n*H$_2$ increases the exergy by around 0.72kWh kg$^{-1}$ in case the *p*H$_2 \rightarrow$ *o*H$_2$ conversion is utilized.

### Synergetic effects of coupling rCEUS and FCS

#### FCS rejected heat reduction, rejected heat utilization and power demand reduction using a rCEUS

Three of the rCEUS evaluation parameters introduced in “Coupling the output powers of FCS and rCEUS” are shown in Fig. 5 for varying FCS efficiencies, FCS operating temperatures and exemplary hydrogen states of different cryogenic hydrogen storage options (LH$_2$, sLH$_2$, CcH$_2$). The top row in Fig. 5 shows $\Pi _P$, the share of the system’s reference output power that can be supplied by the rCEUS. The second row shows the fraction of FCS rejected heat utilizable by a rCEUS, $\Pi _{{\dot{Q}}}$. The third row shows the overall percentage by which the FCS rejected heat to ambient can be reduced when integrating a rCEUS, including the reduced amount of rejected heat due to the FCS power demand reduction and the utilization of parts of the remaining amount. As for the hydrogen composition, *e*H$_2$ is chosen to show the benchmark rCEUS performance, because the *p*H$_2 \rightarrow o$H$_2$ conversion is included.

One can observe that

- FCS rejected heat to ambient reductions of $\Phi _{{\dot{Q}}}$ = 40% ... 67% are possible for FCS efficiencies in the range of $\eta _{\mathrm {FCS}}$ = 30% ... 70% for conceivable low-temperature polymer electrolyte membrane (PEM) stack operating temperatures.
- $\Phi _{{\dot{Q}}}$ has a flat minimum at around $\eta _{\mathrm {FCS}} =$ 40%, so higher FCS rejected heat to ambient reductions are obtained for both higher and lower FCS efficiencies than 40%.
- the rCEUS can supply $\Pi _P =$ 14% ... 31% of the overall system output power demand, depending on FCS efficiency.
- the largest $\Pi _{P}$, $\Pi _{{\dot{Q}}}$ and $\Phi _{{\dot{Q}}}$ of the compared storage states are obtained by liquid hydrogen at possibly low pressure. This is because of the increasing vaporization enthalpy combined with the also increasing Carnot efficiency.
- in the range of conceivable FCS operating temperatures, the influence of the FCS operating temperature on $\Pi _{{\dot{Q}}}$ and $\Pi _P$ and therefore $\Phi _{{\dot{Q}}}$ is negligible. This is because the ratio of hot and cold temperatures does not change much in the range of conceivable FCS operating temperatures for the considered cryogenic hydrogen temperatures.

#### Work and heat involved in each hydrogen conditioning step using a rCEUS

Figure 6 shows how much each hydrogen processing step contributes to the total amount of rCEUS output power and utilizable FCS rejected heat flux for different hydrogen storage options and $p\mathrm {H}_2$–$o\mathrm {H}_2$ mixtures. The maximally obtainable rCEUS output power and utilizable heat flux is independent of the process path, but the shares belonging to each process step, are not. Exemplarily, the following hydrogen conditioning steps are shown, beginning from the storage state: isobaric heat supply to FCS operating temperature and a subsequent isothermal expansion to FCS operating pressure. One can observe that

- when considering hydrogen storage in the vapor-liquid region, evaporation and sensible gas heating are the dominant processes in terms of obtainable work and utilizable FCS rejected heat.
- lower liquid pressures increase heat and work of evaporation due to increased vaporization enthalpy and Carnot efficiency.
- for both maximally obtainable work $w_{\mathrm {rCEUS,out}}$ and utilizable rejected FCS heat $q_{\mathrm {rCEUS,in}}$, *e*H$_2$ > *p*H$_2$ > *n*H$_2$. This is because for a certain pressure the $c_p$ of $p\mathrm {H}_2$ is larger than that of $o\mathrm {H}_2$ in the relevant temperature range.
- for CcH$_2$, the process step accounting for the largest fraction of obtainable work and utilizable heat changes during operation between sensible gas heating and (isothermal) expansion.
- for CcH$_2$ $w_{\mathrm {rCEUS,out}}$ and $q_{\mathrm {rCEUS,in}}$ are the smallest of the compared storage options, because the evaporation as heat sink at constant low temperature combined with the high Carnot efficiency does not exist.

### Maximally obtainable work and corresponding amounts of heat of the *p*H$_2 \rightarrow o$H$_2$ spin state conversion

Figure 7 shows the specific energies of the rCEUS (heat in, heat out, work out) corresponding to the *p*H$_2 \rightarrow o$H$_2$ conversion. Heat at reference temperature $T_{\mathrm {FCS}} = {353}\;K$ is supplied to the rCEUS. The rCEUS heat sink is the endothermic spin state conversion during temperature increase from initially 20K-*e*H$_2$ vapor to 353K-*e*H$_2$ shown in the top image. The conversion is performed continuously, i.e. the *e*H$_2$ composition is reached at every temperature. The temperature dependent *p*H$_2$ and *o*H$_2$ mole fractions in *e*H$_2$ and the corresponding Carnot efficiencies are shown in the middle and bottom image. Only a continuous instead of a step-wise conversion during hydrogen temperature increase enables the maximum work to be obtained and the maximum FCS rejected heat utilization. This is, because a step-wise conversion can not reach Carnot efficiency. From Fig. 7 one can observe that

- the maximally obtainable work from converting 20K-*e*H$_2$ continuously to 353K-*e*H$_2$ is $w_{p\mathrm {H}_2 \rightarrow o\mathrm {H}_2,\mathrm {max}} \approx {0.72} \; \text {kWh} \text {kg}^{-1}$ using heat at $T_{\mathrm {FCS}} = {353}\;K$ reference temperature. This work is practically independent of pressure (compare Fig. 3, bottom right image).
- in the demonstrated case the spin state conversion utilizes FCS rejected heat with an overall efficiency of around $\eta _{p\mathrm {H_2} \rightarrow o\mathrm {H_2}} := \frac{w_{p\mathrm {H}_2 \rightarrow o\mathrm {H}_2,\mathrm {max}}}{q_{\mathrm {rCEUS, in,} p\mathrm {H}_2 \rightarrow o\mathrm {H_2}}} =$ 84%. In other words: ideally, around 119% of the conversion energy can be carried off the FCS coolant circuit.
- the conversion allows to carry off around 0.86 kWh kg$^{-1}$ FCS rejected heat at best.
- most conversion energy can be recovered already at cryogenic temperatures. For example: 95% of the maximally obtainable work is already obtainable once hydrogen has reached around 97 K.
- the conversion has a slightly larger energy demand as low-pressure evaporation. Nevertheless, the conversion yields a lower FCS rejected heat reduction than evaporation. This is, because the conversion is subject to decreasing Carnot efficiencies due to the *e*H$_2$ composition’s temperature dependency.

### Comparison of FCS rejected heat reductions using a rCEUS and conventional HXs

The state-of-the-art method to thermally couple a cryogenic storage system with a FCS is to use FCS coolant to heat hydrogen in HXs. The rCEUS in comparison is not only able to carry off a significantly larger amount of rejected heat, but to also utilize it to save fuel and reduce the amount of FCS heat rejected to begin with. The ratio of FCS rejected heat reduction using a rCEUS and FCS rejected heat reduction using HXs, $\Phi _{{\dot{Q}}}/\Pi _{{\dot{Q}}, \mathrm {HX}}$, is shown in Fig. 8 for varying FCS efficiencies and operating temperatures for the exemplary operating states of the storage forms LH$_2$, sLH$_2$ and CcH$_2$.

One can observe that depending on FCS efficiency, a rCEUS can reduce FCS rejected heat to ambient by a factor of 4.5–8 compared to conventional HXs. This ratio decreases with increasing FCS efficiency as a consequence of Eqs. (32) and (33). FCS operating temperatures have a negligible impact on the ratio. FCS efficiency is the dominant dependency.

## Summary and conclusion

A thermodynamically perfect exergy utilization system for different storage forms of cryogenic hydrogen in a fuel cell system was investigated in terms of fuel cell system thermal management and power demand reduction. The exergy utilization system uses fuel cell system rejected heat as heat supply and hydrogen as heat sink to run reversible heat engine processes and generate electrical power supporting the fuel cell system. FCS heat rejection to ambient is thereby drastically reducible compared to conventional heat exchangers used for hydrogen conditioning. Methods and simple analytical correlations to assess the system’s performance are presented and synergetic effects for thermal management are quantified: it is found that, at best, the fuel cell system rejected heat can be reduced by 40–67% and its power demand can be reduced by around 14–31% depending on fuel cell system efficiency. The influence of the stack operating temperature is negligible for low-temperature cells.

Implications and future recommendations are twofold: on the CEUS level (1), suitable real CEUS topologies, their processes, components and working fluids should be identified and optimized. This can be used to estimate real CEUS topology efficiency, output power, mass and volume. On the system level (2)—e.g. aircraft, heavy-duty trucks or other systems based on fuel cells and cryogenic hydrogen—synergies with several subsystems should be investigated. Such subsystems include:

- the thermal management system: smaller and/or lighter components, such as heat exchangers are conceivable since less heat has to be transferred to the ambient when using a CEUS
- cryogenic hydrogen tanks: smaller and lighter tanks are conceivable given the decreased total hydrogen demand
- fuel cell stacks: higher stack efficiency due to operation at lower current density at constant stack size or lighter and smaller stacks at constant efficiency are conceivable given the reduced stack power demand
- electric components: efficiencies could be increased by utilizing effects of superconductivity enabled at cryogenic hydrogen temperatures. This however requires sacrificing parts of the CEUS’s heat sink.

Deploying a CEUS in mobile fuel cell system enables several new technology options and overall system architectures such that a considerable increase in overall system fuel efficiency may be achievable.

## Data Availability

All presented data and the MatLab implementation of the calculation scheme are available upon reasonable request from the corresponding author.

## Roman letters

$c_{p}$: Specific isobaric heat capacity ($\mathrm{J\; kg}^{-1} \mathrm{K}^{-1}$)
*e*: Specific energy ($$\mathrm{J \; kg}^{-1}$$)
*h*: Specific enthalpy ($$\mathrm{J\; kg}^{-1}$$)
$\hslash$: Reduced Planck constant, $\hslash = 1.0546 \times 10^{-34}$ Js (Js)
*I*: Moment of inertia ($\mathrm{kg} \; \mathrm{m}^{2}$)
$$k_{\mathrm {B}}$$: Boltzmann constant, $$k_{\mathrm {B}} = 1.3806 \times 10^{-23} \text {JK}^{-1}$$ ($$\mathrm{JK}^{-1}$$)
$${\dot{m}}$$: Mass flow ($$\mathrm{kg\;s}^{-1}$$)
*n*: Amount of substance (mol)
*P*: Power ($$\mathrm{J \; s}^{-1}$$)
*p*: Pressure (Pa)
$${\dot{Q}}$$: Heat flux ($$\mathrm{J \; s}^{-1}$$)
*q*: Specific heat ($$\mathrm{J\; kg}^{-1}$$)
*s*: Specific entropy ($$\mathrm{J \; kg}^{-1} \mathrm{K}^{-1}$$)
*T*: Temperature (K)
*w*: Specific work ($$\mathrm{J \; kg}^{-1}$$)
$w_{\mathrm {H_2}}$: Lower heating value of hydrogen, $w_{\mathrm {H_2}} = 120 \times 10^{6} \text {J} \; \text {kg}^{-1}$ ($\mathrm{J \; kg}^{-1}$)

## Greek letters

$$\eta$$: Efficiency (–)
$$\Theta$$: Rotational constant (K)
$$\xi$$: Mass fraction (–)
$$\rho$$: Density ($$\mathrm{kg} \; \mathrm{m}^{-3}$$)

## Subscripts

evap: Vaporization
ex: Exergy
rCEUS: Reversible cryogenic exergy utilization system
FCS: FCS hydrogen inlet state, reference state (reservoir)

## Superscripts

($${\overline{\cdot }}$$): Mean
($\tilde{\cdot }$): FCS and rCEUS are coupled
(${\hat{\cdot }}$): FCS and rCEUS are not coupled

## Abbreviations

CcH$_2$: Cryo-compressed hydrogen
*e*H$$_2$$: Equilibrium hydrogen
FC: Fuel cell
FCS: Fuel cell system
H$$_2$$: Molecular hydrogen
LH$_2$: Liquid hydrogen
*n*H$$_2$$: Normal hydrogen
*o*H$$_2$$: Orthohydrogen
*p*H$$_2$$: Parahydrogen
sLH$_2$: Subcooled liquid hydrogen
